# Quantification of irrigated lesion morphology using near-infrared spectroscopy

**DOI:** 10.1038/s41598-021-99725-8

**Published:** 2021-10-11

**Authors:** Soo Young Park, Rajinder Singh-Moon, Haiqiu Yang, Deepak Saluja, Christine Hendon

**Affiliations:** 1 Department of Electrical Engineering, Columbia University, 500 West 120th Street, New York, NY 10027 USA; 2 Department of Medicine (Cardiology), Columbia University College of Physicians and Surgeons, 630 W. 168th St, New York, NY 10032 USA

**Keywords:** Cardiology, Engineering, Optics and photonics

## Abstract

There are currently limited means by which lesion formation can be confirmed during radiofrequency ablation procedures. The purpose of this study was to evaluate the use of NIRS-integrated RFA catheters for monitoring irrigated lesion progression, ex vivo and in vivo. Open-irrigated NIRS-ablation catheters with optical fibers were fabricated to sample tissue diffuse reflectance. Spectra from 44 irrigated lesions and 44 non-lesion sites from ex vivo swine hearts (n = 15) were used to train and evaluate a predictive model for lesion dimensions based on key spectral features. Additional studies were performed in diluted blood to assess NIRS signatures of catheter-tissue contact status. Finally, the potential of NIRS-RFA catheters for guiding lesion delivery was evaluated in a set of in vivo pilot studies conducted in healthy pigs (n = 4). Model predictions for lesion depth (*R* = 0.968), width (*R* = 0.971), and depth percentage (*R* = 0.924) correlated well with measured lesion dimensions. In vivo deployment in preliminary trials showed robust translational consistency of contact discrimination (P < 0.0001) and lesion depth parameters (< 3% error). NIRS empowered catheters are well suited for monitoring myocardial response to RF ablation and may provide useful intraprocedural feedback for optimizing treatment efficacy alongside current practices.

## Introduction

In radiofrequency ablation (RFA) of arrhythmias, long-term treatment outcome is predicated in part on effective lesion delivery to interrupt electrical conduction and/or eliminate focal triggers. Lesion dimensions, such as depth and width, are critical factors for a durable response^1–4^. Intraprocedural lesion assessment is limited, and areas of incomplete treatment can permit conduction recovery and poor response to ablation^5–7^. There is a push towards the development and use of lesion size predictors to assess lesion sufficiency^2,3,8,9^, driven by the correlation of ablation parameters (e.g power, contact force, time, impedance) with average lesion size. In addition, approaches are being pursued for the direct visualization of injury to the cardiac wall^10–18^. A real-time, integrated assessment of lesion sufficiency derived from estimates of tissue necrosis could both enable feedback-mediated titration of RF energy during ablation, and serve as a complement to current lesion set validation tools.

In recent years, a number of optical technologies have been proposed for direct evaluation of the extent of necrotic tissue following RFA. Previously, optical coherence tomography (OCT) has been adopted to directly visualize myocardial tissue and discern tissue treatment with high spatial resolution^19–21^. OCT-integrated catheters enabled real-time imaging feedback to identify tissue structures and direct monitoring of RF lesion formation, ex vivo^19,22–24^ and in vivo^15,25^. Recently, acute necrotic areas in atrial tissue were identified using hyperspectral imaging system based on ultraviolet-excited tissue autofluorescence^11,26–28^. Another promising technique, near-infrared spectroscopy (NIRS), uses broadband light to interrogate tissue volumes deeper than OCT and ultraviolet-excited tissue autofluorescence. NIRS can be used to characterize important physiological properties from spectral signatures. In prior work, several groups, including ours, have shown the ability of NIRS for tracking non-irrigated lesion depth in ex vivo myocardial slabs^29–32^. Though hopeful demonstrations, little to no work has been shown for directly monitoring of irrigated lesions, which constitute the vast majority of lesions delivered clinically today^33–35^. Thus, the objective of this work was to explore the feasibility of NIRS for real time assessment of lesion dimensions created with an irrigated RFA catheter. In this study, we developed a NIRS-integrated open-irrigation ablation catheter and an algorithm for assessing lesion dimensions based on key features derived from NIRS measurements. Using this model, we demonstrate real-time tracking of irrigated lesion delivery in both an ex vivo and in vivo swine model.

## Methods

### Optical catheter construction

**Figure 1:**
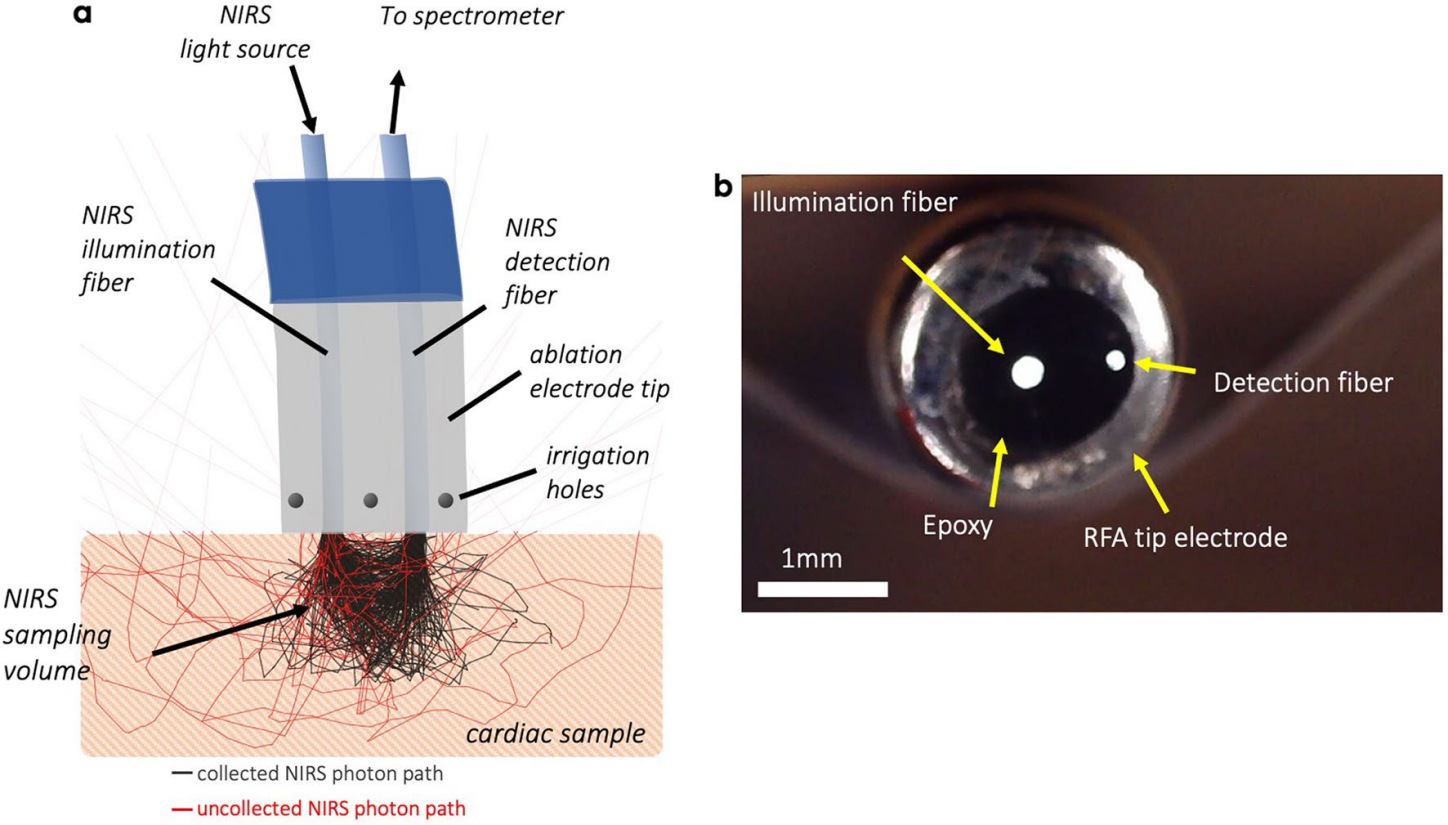
Combined NIRS radiofrequency ablation catheter. (**a**) Cartoon illustrating the concept of RF ablation catheters incorporating NIRS measurement and its salient features. (**b**) Finished catheter construction distal end showing embedded optical fibers for NIRS measurement at the tip electrode.

Catheters combining NIRS fibers and irrigation holes were used to monitor spectral changes in tissue throughout lesion delivery. A schematic diagram of the salient features is depicted in Fig. 1. To fabricate these, 7-F, 3.5 mm open-tip irrigated Celsius Thermocool RFA catheters (DI7TCDLRT, Biosense Webster, Diamond Bar, CA) were modified to accept two multi-mode optical fibers within the inner saline channel. From benchtop testing, our catheters were able to sustain an irrigation flow rate of 17 ml/min^34,36^. The fiber distal ends were terminated at the catheter tip to permit real-time optical sampling of the RFA treatment zone during energy delivery. A detailed description of our NIRS system has been previously described^12,31^. Briefly, to carryout NIRS measurements, one fiber delivered broadband light (HL-2000HP, Ocean Optics Inc, Dunedin, FL) to the tissue while the remaining fiber collected tissue-backscattered light to a spectrometer (600–1000 nm) (C9405CB, Hamamatsu, Bridgewater, NJ). Measurements were recorded on a laptop computer.

### In vitro experimental protocol

The experimental protocol for real-time measurement of lesion formation is similar to that used previously^12^. Fresh whole hearts (n = 15) from healthy swine were acquired within 24 h of sacrifice (Green Village Packing Co., Green Village, NJ). Right ventricular wall segments were excised and submerged in a flowing bath of phosphate buffered saline (37 °C). The manual unipolar mode was utilized for all ablations. Power and duration settings were varied between 20–30 W and 15–60 s, respectively, to create a range of lesion sizes, where the saline irrigation settings was maintained at 10 ml/min. Optical measurements were initiated just prior to the onset of ablation at baseline and were recorded continuously throughout at 50 Hz until 20 s post ablation. Following optical data acquisition, lesions were bisected and submerged in 1% 2,3,5-Triphenyl-2H-tetrazolium chloride (TTC) vital stain for 40 min at room temperature to assess the extent of tissue necrosis. TTC-stained samples were digitized using a digital camera. The maximal lesion width (LW), central lesion depth (LD) and percentage of relative treatment depth (LD%) were manually segmented from TTC stained cross-sections. The lesion boundaries were chosen based on the intersection between the stained (red) and unstained (white) tissue. A total of 88 lesions were created, ranging from 0–6 mm in LD and 0–11 mm in LW.

### In vivo experimental protocol

Preliminary assessment of in vivo optical guidance of RFA was performed in a total of four pigs within the right ventricle. Healthy adult swine underwent endotracheal intubation for mechanical ventilation of mixed gas anesthesia isoflurane 1–5% for sedation. Physiological parameters including core body temperature, EKG, oxygen saturation, end-tidal CO_2_, heart rate, and respiratory rate were monitored throughout the course of the experiments. Percutaneous catheter navigation was performed under ultrasound and contrast-enhanced fluoroscopic guidance. Unipolar, power-controlled ablation was performed with power varied between 10 and 30 W for durations between 30 and 60 s. Saline irrigation was maintained at 10 ml/min as we experienced lesions created with higher flow rates more commonly induced ventricular arrhythmias in the swine. Following endocardial lesion delivery, pigs were euthanized, and a thoracotomy was done for additional epicardial ablation and measurements for validation. Hearts were then harvested and immediately placed on ice for further assessment of tissue injury. All animal studies conformed to the Guide for the Care and Use of Laboratory Animals and were approved by Columbia University’s Institutional Animal Use and Care Committee. Experiments were conducted in accordance with the recommendations in the Animal Research: Reporting In Vivo Experiments (ARRIVE).

### Lesion assessment

**Figure 2:**
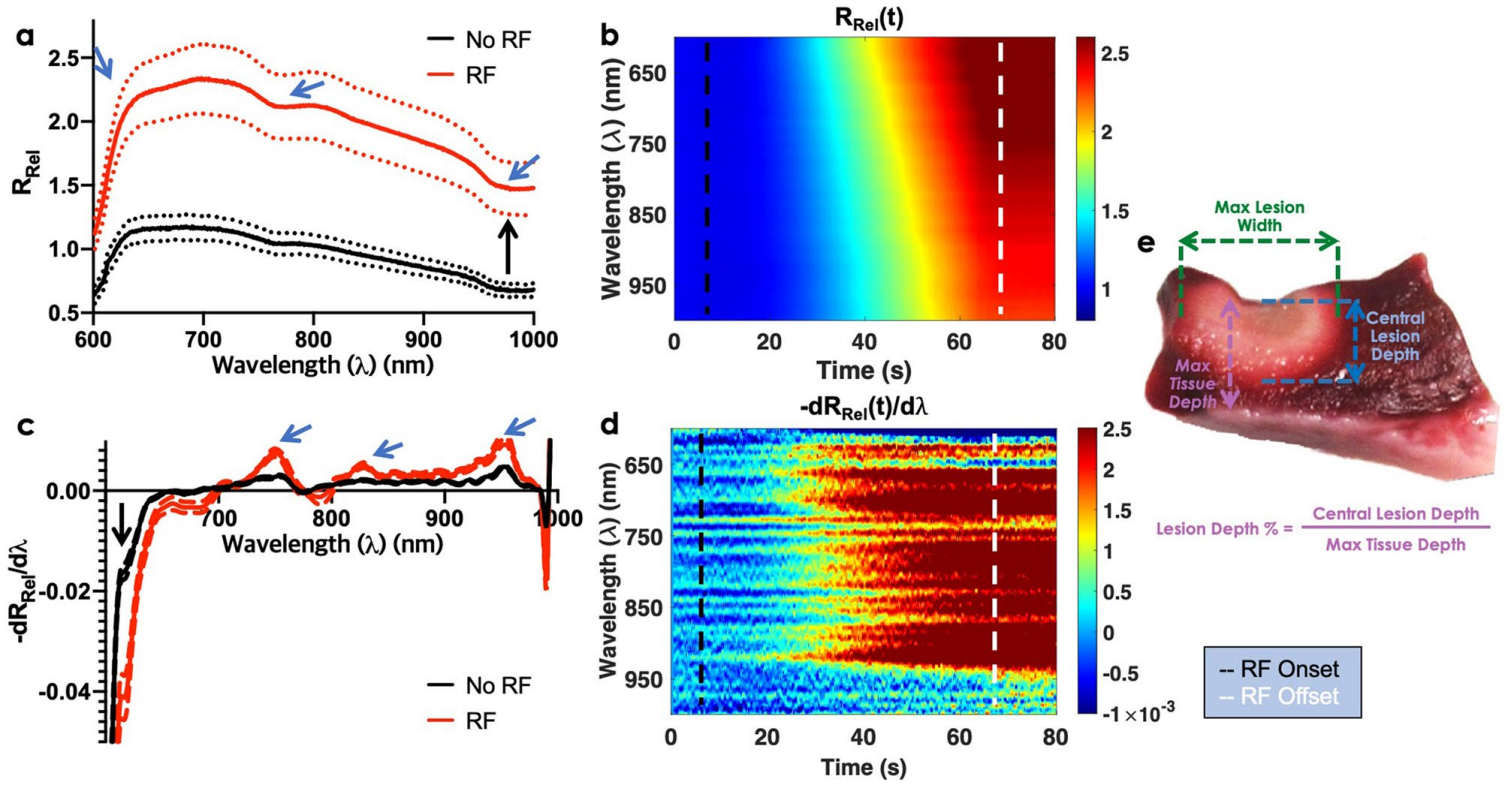
Exemplary real-time monitoring of lesion formation using NIRS. (**a**) Spectral changes in measured relative reflectance (*R*_*Rel*_) in response to RF energy (n_RF_ = 44, n_noRF_ = 44). Two main groups of changes were observed: an increase in the mean spectral amplitude or offset (black arrow) and pronounced changes in spectral morphology (blue arrows). Dashed lines (standard deviation). Bold line (mean) (**b**) Temporal changes in measured relative reflectance in response to RF ablation. (**c**) The first derivative of the *R*_*Rel*_ curve, normalized at 650 nm, to further emphasize changes in spectral shape. With RF onset, *R*_*Rel*_ near 600–700 nm decreased (black arrow), but *R*_*Rel*_ in the 740–760 nm and 840–960 nm increased. (**d**) Temporal changes in the first derivative of the *R*_*Rel*_ curve. (**e**) Corresponding lesion stained with TTC to reveal the extent of RFA damage. The maximum width and central lesion depth were derived from digitized images as shown. Black dashed lines (RF ablation onset). White dashed lines (RF ablation offset).

At each ablation location, maximum lesion diameter and central lesion depth were determined from TTC stained cross sections (Fig. 2e). Additionally, lesion depth percentage was derived by measuring the amount of ablated myocardial tissue relative to the total tissue thickness. Prior studies showed that lesion insufficiency and gaps are major factors for arrhythmia resurgence^37,38^. However, overtreatment can potentially cause thermal injury to surrounding tissues (for example, the esophagus and coronary vasculature)^39^, which can be life threatening. Therefore, LD% provides an independent parameter for clinicians to titrate RF energy dose and avoid possible collateral tissue damage.

### NIRS signal processing and feature extraction

All NIRS measurements first underwent calibration to isolate the tissue signal and remove the wavelength-dependent output and sensor sensitivities of the light source and spectrometer, respectively^31,40^. This is accomplished by taking measurements on a spectrally flat diffuse reflector for white balance, and on an optically stable phantom for normalization. Completing this process converts raw spectrometer readings into relative reflectance (*R*_*Rel*_), a tissue property and optical-configuration dependent quantity that can be compared while accounting for day-to-day variations in lamp output. Figure 2a shows mean *R*_*Rel*_ spectra of RF delivered tissue and that of unablated myocardial tissue. RF ablation *R*_*Rel*_ shows a distinct local minimum near 760 nm and an elevation in the 600–700 nm range. In Fig. 2b, spectro-temporal *R*_*Rel*_ responses increase during RF energy delivery. This can be attributed to dominant scattering effect in ablated tissue^30,31,41,42^. Negative derivative of *R*_*Rel*_ ($- R_{Rel}^{^{\prime}}$) revealed two local maxima near 760 nm and 960 nm (Fig. 2c), which can be associated by absorption changes during ablation^42^. Overall, mean RF delivered $- R_{Rel}^{^{\prime}}$ values in the 700–950 nm range was higher compared to no RF tissue. Based on these spectral differences, we established 9 parameters called lesion optical index (LOI). These parameters are described below:

1 $$LOI_{1} = \mathop \sum \limits_{{ \wedge_{1} }}^{{ \wedge_{2} }} R_{Rel} (\lambda ),\;\;[ \wedge_{1} , \wedge_{2} ] = \left[ {600\;{\text{nm}},\;1000\;{\text{nm}}} \right]$$

2 $$LOI_{2} = \frac{{R_{Rel} \;(700\;{\text{nm}})}}{{R_{Rel} \;(622\;{\text{nm}})}}$$

3 $$LOI_{3,4,5,6} = \mathop \sum \limits_{{ \wedge_{3} , \wedge_{4} , \wedge_{5} , \wedge_{6} }} \left[ {R_{Rel}^{^{\prime}} \left( \lambda \right) - R_{Rel}^{^{\prime}} (650\;{\text{nm}})} \right],\quad \begin{array}{*{20}c} { \wedge_{3} = \left[ {600\;{\text{nm}},\;1000\;{\text{nm}}} \right]} \\ { \wedge_{4} = \left[ {600\;{\text{nm}},\;700\;{\text{nm}}} \right]} \\ { \wedge_{5} = \left[ {700\;{\text{nm}},\;800\;{\text{nm}}} \right]} \\ { \wedge_{6} = \left[ {800\;{\text{nm}},\;1000\;{\text{nm}}} \right]} \\ \end{array}$$

4 $$LOI_{7} = \frac{{R_{Rel}^{^{\prime}} (601\;{\text{nm}})}}{{R_{Rel}^{^{\prime}} (609\;{\text{nm}})}}$$

5 $$\begin{gathered} LOI_{8} = R_{Rel}^{^{\prime}} (601\;{\text{nm)}} - R_{Rel}^{^{\prime}} (609\;{\text{nm}}) \end{gathered}$$

6 $$LOI_{9} = \left\{ {\begin{array}{*{20}c} { - 1, R_{Rel}^{^{\prime}} (601\;{\text{nm}}) - R_{Rel}^{^{\prime}} (609\;{\text{nm}}) < 0} \\ {1, R_{Rel}^{^{\prime}} (601\;{\text{nm}}) - R_{Rel}^{^{\prime}} (609\;{\text{nm}}) \ge 0} \\ \end{array} } \right.$$

We introduce LOI_1_, mean value of *R*_*Rel*_, to highlight increase reflectance in RF treated tissue (Fig. 2a). LOI_2_ reflects increases in RF treated reflectance near 700 nm by computing the steepness of the line between 622 and 700 nm. LOI_3–6_ are areas under the $- R_{Rel}^{^{\prime}}$ curve at various wavelength ranges to capture local maxima and minima in $- R_{Rel}^{^{\prime}}$ (Fig. 2c,d). LOI_7-9_ are calculated based on abrupt surge observed between 600 and 609 nm in treated tissue.

### Lesion size estimation model

Ex vivo experiments were conducted to collect a large number of RF lesions and build a mathematical model for lesion size approximation. We employed a gaussian process regression model^43^ using the LOIs described in Eqs. (1)–(9) as input variables and lesion dimensions determined from digitized TTC measurements. Lesion depth, width and depth percentage for spectra collected from catheter-tissue contact and untreated tissue were set to zero. A gaussian process regression model is a supervised learning model where the input and the output are mapped from the training data. It is a non-parametric Bayesian modelling technique that combines prior distribution of known observed data to derive posterior predictive distributions for unknown future data. The relationship between the output and input values are restricted to a specific gaussian form. It has been shown to capture complex nonlinear relationships between variables. All processing was performed in MATLAB (The Mathworks Inc., Natick, MA).

### Statistical analysis

Analysis of variance with multiple comparison test was used to evaluate significance in the extracted 9 LOI features over tissue contact and extent of tissue injury. Agreement between lesion dimensions and extracted features were quantified using Pearson's correlation coefficient. P-values less than 0.05 were deemed significant. Prism 8 (Graphpad Software, San Diego, California) was used for statistical analyses.

## Results

### Ex vivo contact analysis

**Figure 3:**
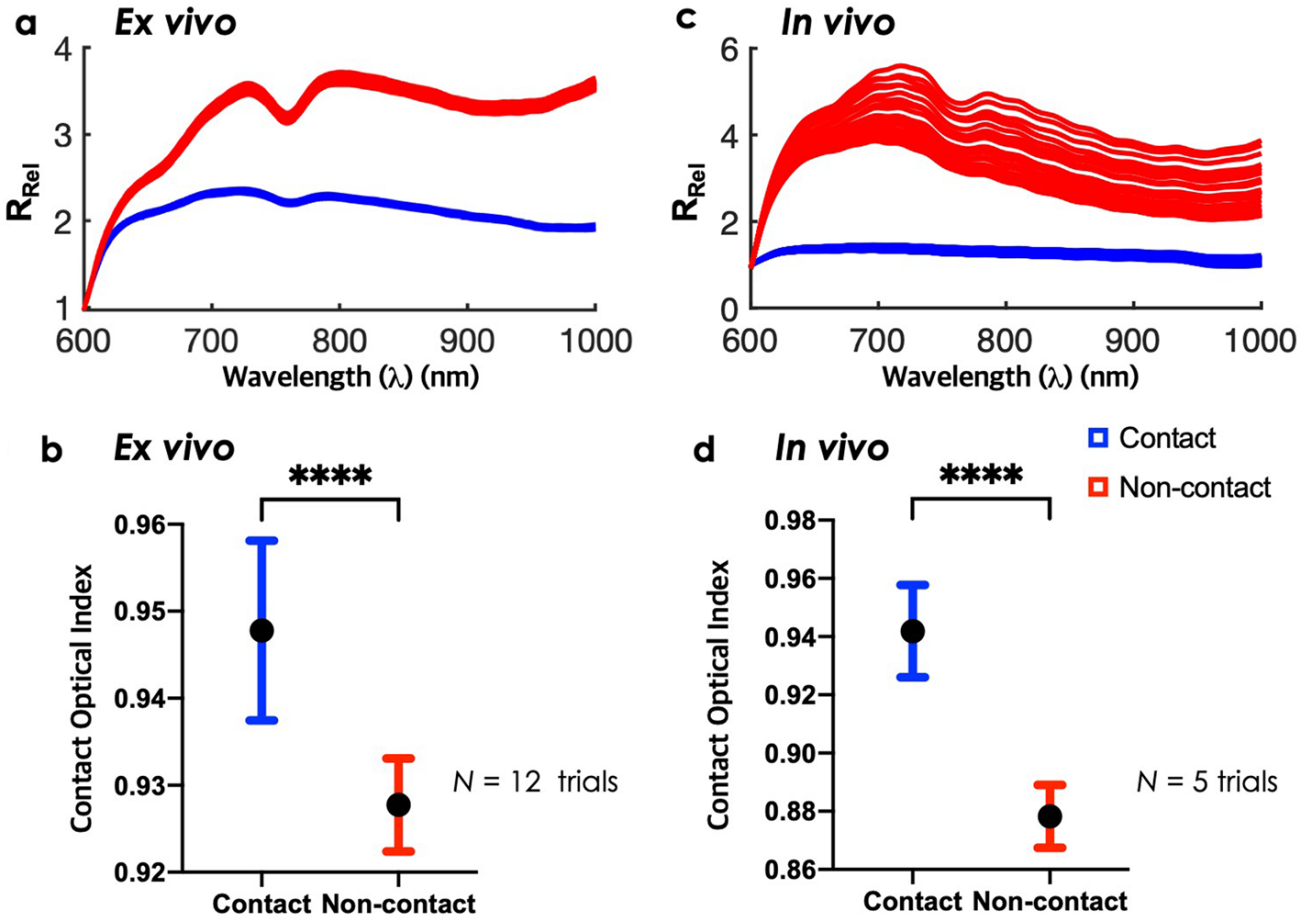
Catheter probe contact assessment ex vivo and in vivo. (**a**) Represents *R*_*Rel*_ spectra of ex vivo contact (red) and non-contact (blue) measurements for 10 s. Black dashes denote contact optical index (COI) wavelengths. COI is described as follows: *R*_*Rel*_ (764 nm)/*R*_*Rel*_(730 nm). (**b**) Compares recorded COI between contact and non-contact. Bars are presented as mean and standard deviation (****P < 0.0001). (**c**,**d**) Repeated for in vivo demonstration.

Initial ex vivo contact experiments were performed on swine RV submerged in blood. *R*_*Rel*_ spectra were obtained by normalizing the raw spectra at 600 nm. Figure 3a shows recorded *R*_*Rel*_ spectra during direct catheter contact (blue) on the RV endocardial surface and catheter pulled back from tissue (red) under blood. Data was acquired in 10 s intervals. The amplitude of *R*_*Rel*_ spectra increased when probe was separated from tissue surface. *R*_*Rel*_ measurements floating in blood show a steep increase in the 600–700 nm range and leads to a strong local minimum centered at 760 nm. At 980 nm, we observe a subtle increase. Once the probe made contact with tissue, *R*_*Rel*_ remained relatively monotonic. Based on these differences in spectral morphology, contact optical index (COI) was computed as follows:

7 $$COI = { }\frac{{\left. {R_{Rel} (764nm} \right)}}{{\left. {R_{Rel} (730nm} \right)}}$$

Using COI, contact and non-contact within blood were differentiable as shown in Fig. 3b (n = 12 trials). Statistical analysis showed significant differences between catheter tissue contact and non-contact (P < 0.0001). COI can be tracked in real-time, showing a sharp transition between contact and non-contact (Supplementary Figure 2).

### Ex vivo regression model evaluation

**Figure 4:**
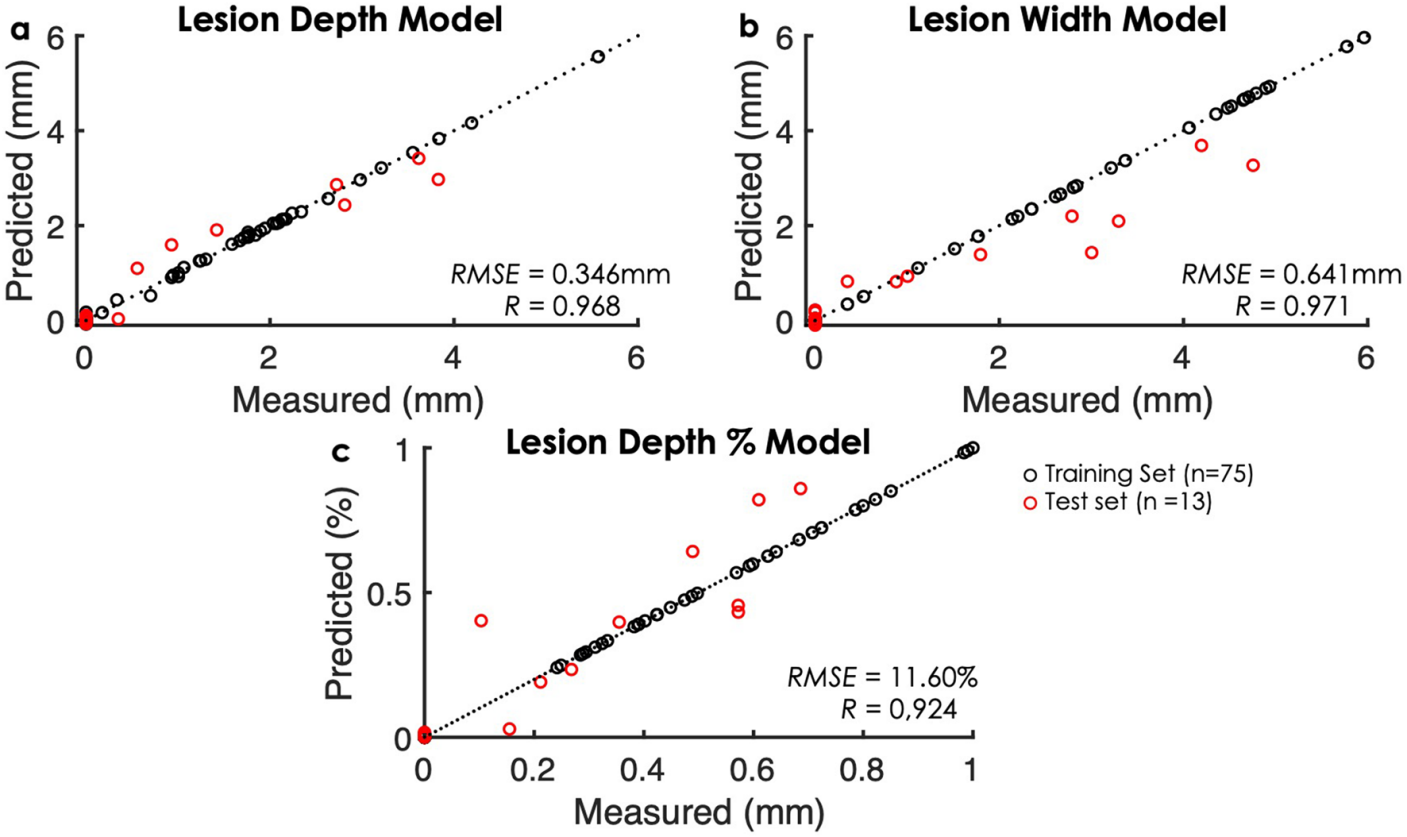
Comparison between model predictions and measured lesion depth (**a**), width (**b**), and depth percentage (**c**) across all samples. Black circles correspond to the evaluations across the full training set which comprises the validation set (n = 75); red circles correspond to test set samples (n = 13).

LOI-based gaussian process models were evaluated by root mean squared error and Pearson’s correlation coefficients (R). All 9 extracted LOI features were included in the models. Figure 4 shows results of the regression models for predicted lesion dimensions to actual values (n_training_ = 75, n_test_ = 13). Pearson’s correlation coefficient values for predicted lesion depth, width, and depth percentage models indicated strong linear relationships with actual measurements from TTC cross-sections (R_LD,LW,LD%_ > 0.9). The root mean square error for predicted lesion depth showed strong prediction agreements within the range of 0.3 mm. The model error for predicted lesion width was higher (~ 0.6 mm) than the model error for lesion depth. This can be potentially attributed to the limited sampling volume in parallel direction and the optical fiber numerical aperture. The numerical aperture for our optical fibers is 0.22, which limits sampling in the x-direction. The error in the lesion depth percentage regression model was 11%. The impact of myocardial tissue thickness must be assessed further to reduce this variability.

### Real-time monitoring of lesion formation

**Figure 5:**
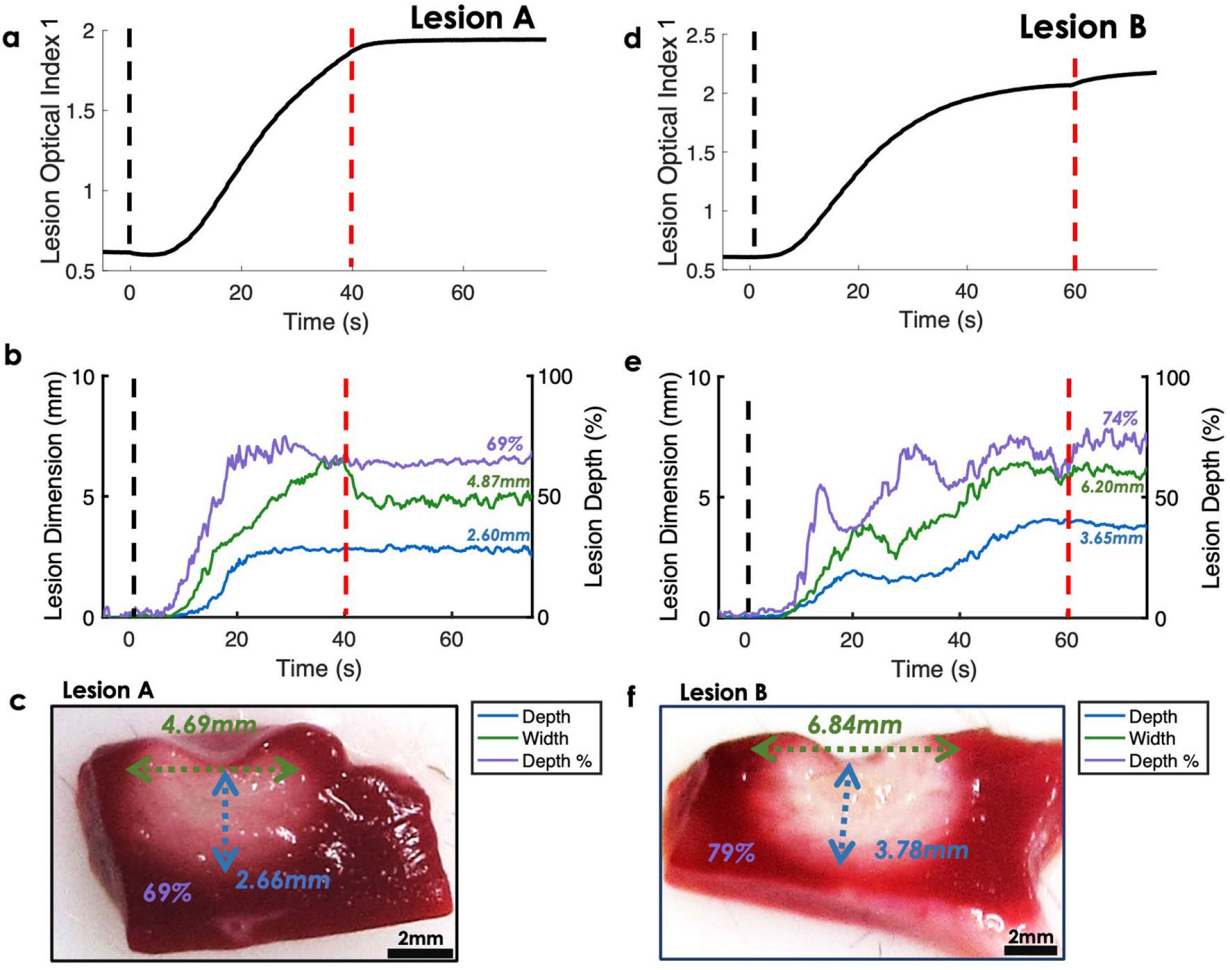
Representative samples for real-time lesion size approximation ex vivo. (**a**,**d**) Show recorded lesion optical index (LOI_1_ = $\sum\nolimits_{{ \wedge_{1} }}^{{ \wedge_{2} }} {}$ *R*_*Rel*_ ($\lambda$), $\left[ { \wedge_{1} ,\; \wedge_{2} } \right]$ = [600 nm, 1000 nm]) changes over time for two different size lesions. Black and red dashes denote the onset and completion. (**b**,**e**) Show the corresponding predictions of lesion depth, width, and depth percentage, along with the resulting TTC stained lesion for comparison in (**c**) and (**f**).

In Fig. 5, we monitored ex vivo lesion formation real-time using our models. As an example, two lesion ablation sites with different lesion dimensions were inputted into gaussian process regression models to observe the dynamic changes of lesion size estimates. LOI_1_ was plotted over NIRS acquisition to demonstrate how it responded to RF ablation. In Fig. 5a,d, LOI_1_ shows a short delay after the start of RF delivery, then a monotonic increase until the end of RF ablation. With RF offset, LOI_1_ stabilized. The model estimations are shown in Fig. 5b,e for lesion A and Lesion B, respectively. A short delay following RF onset was observed in the model prediction and predictions stabilized with RF offset. Overall, predicted lesion dimension values increased with ablation onset and stabilized after ablation offset. Lesion A dimension predictions were close to the actual measurements (Fig. 5c). The discrepancy between the predicted and measured for lesion depth, width, and depth percentage were − 0.06 mm, + 0.18 mm, and 0%, respectively. The resulting model outputs for Lesion B were all within 5% of actual measurements (Fig. 5f). Time traces of all LOIs for ex vivo lesion A are shown in Supplementary Figure 1.

### In vivo pilot experiments

A preliminary in vivo pilot study was conducted in four healthy pigs to evaluate optical monitoring of catheter-tissue contact and RF ablations. During the former, the NIRS-RFA catheter was steered to various locations in the RV under fluoroscopic guidance and established contact was confirmed by bipolar electrograms. Five sets of contact to non-contact transition were acquired. Additionally, five RF lesions were delivered to the RV apex and free wall. Lesion central depth, maximal width and depth percentage measured on TTC stained cross sections ranged from 0.93–3.49 mm, 1.65–9.57 mm, and 18.1–100%, respectively.

**Figure 6:**
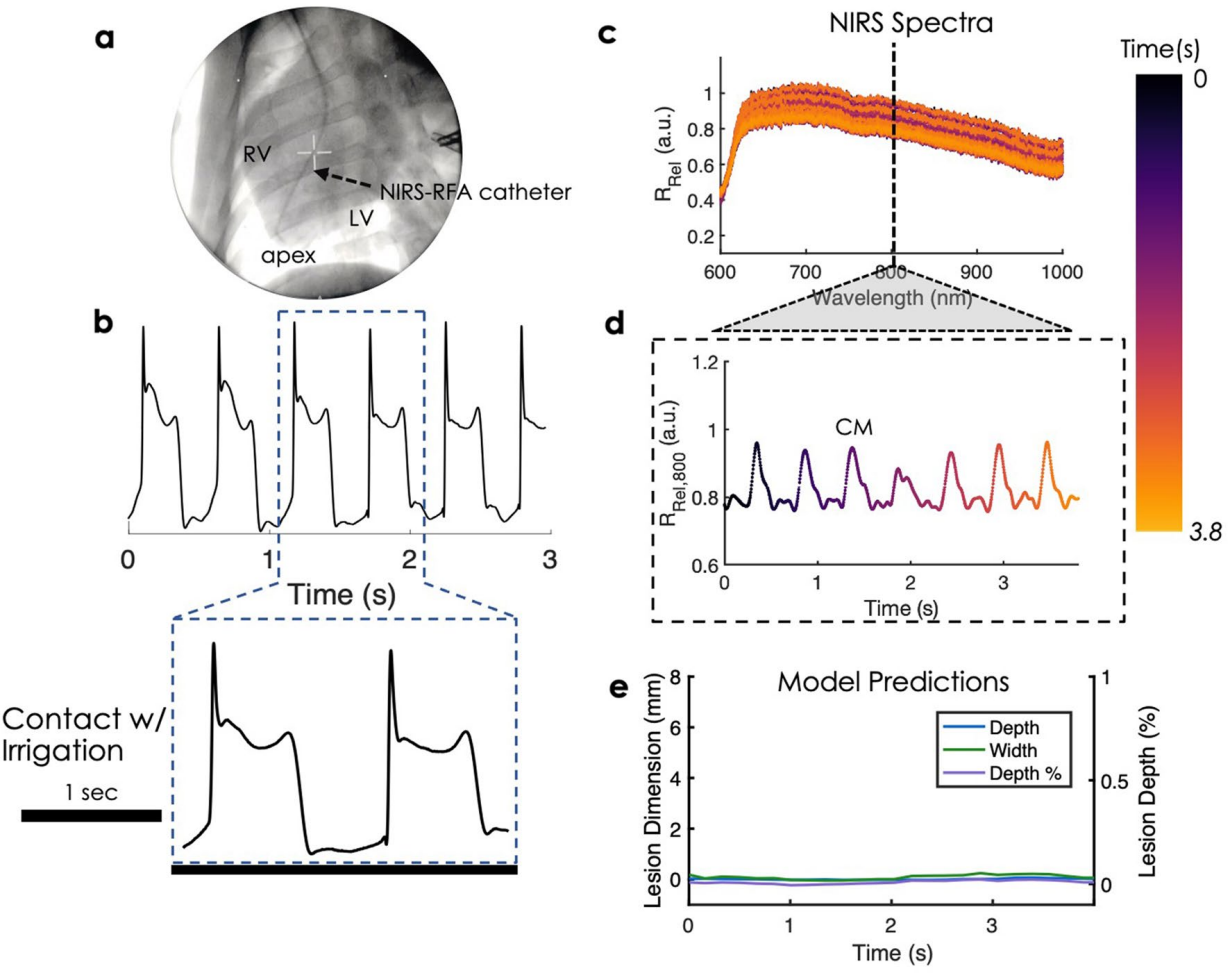
In vivo deployment of NIRS-RFA catheter. (**a**) Shows NIRS-RFA catheter placement at the apex under fluoroscopic guidance. (**b**) Shows bipolar electrogram measurements indicating good catheter contact with tissue. (**c**) Shows corresponding temporal fluctuations of *R*_*Rel*_ spectra over the cardiac cycle. (**d**) Shows amplitude variations at 800 nm reflecting a similar periodicity to the bipolar electrogram. *CM* cardiac motion. (**e**) Shows the corresponding gaussian process regression model outputs with relatively stable predictions under cardiac motion of near zero values for untreated, normal tissue.

First, contact optical indices were derived from spectral features which distinguished catheter-tissue contact on ex vivo swine specimen. Afterwards, we verified that similar spectral changes were observed in vivo. Figure 6a shows a fluoroscopy image of NIRS-RFA catheter lodged in the apex inside the RV, in vivo. Bipolar electrogram measurements confirm adequate catheter tip contact with tissue (Fig. 6b). *R*_*Rel*_ spectra fluctuated up and down when catheter tip was placed on the RV (Fig. 6c). To demonstrate this further, 800 nm was selected to show oscillations in *R*_*Rel*_ signal during tissue contact and confirmed similar periodicity reflected in the bipolar electrogram (Fig. 6d and Supplementary Figure 3). Figure 3c compares NIRS spectra acquired when the probe is in contact with tissue versus suspended in blood. The observed spectral changes were consistent with ex vivo measurements. When the catheter was in direct contact with endocardial surface, *R*_*Rel*_ spectra were relatively flat in shape and showed little variance over time (Fig. 3c). When the catheter tip was pulled back from the tissue, *R*_*Rel*_ spectra measurements revealed strong absorption near 760 nm and high oscillations. The local minimum at 760 nm can be attributed to the increased light absorption due to direct exposure to hemoglobin (Hb)^44^. The greater temporal variance in *R*_*Rel*_ spectra may be attributable to active sampling of circulating blood during the cardiac cycle. Using the contact optical index, statistical analysis revealed significant differences between contact and non-contact in vivo (P < 0.0001) (Fig. 3d). Hence, probe contact and non-contact in vivo could be successfully differentiated by spectral markers from NIRS. LOIs derived from spectra taken during tissue contact were fed into our models. The prediction outputs in Fig. 6e were close to zero and showed stable predictions under cardiac motion for all three parameters (LD, LW, LD%). Next, we verified that spectral changes occur in endocardial lesion delivery and monitored lesion progression, in vivo.

**Figure 7:**
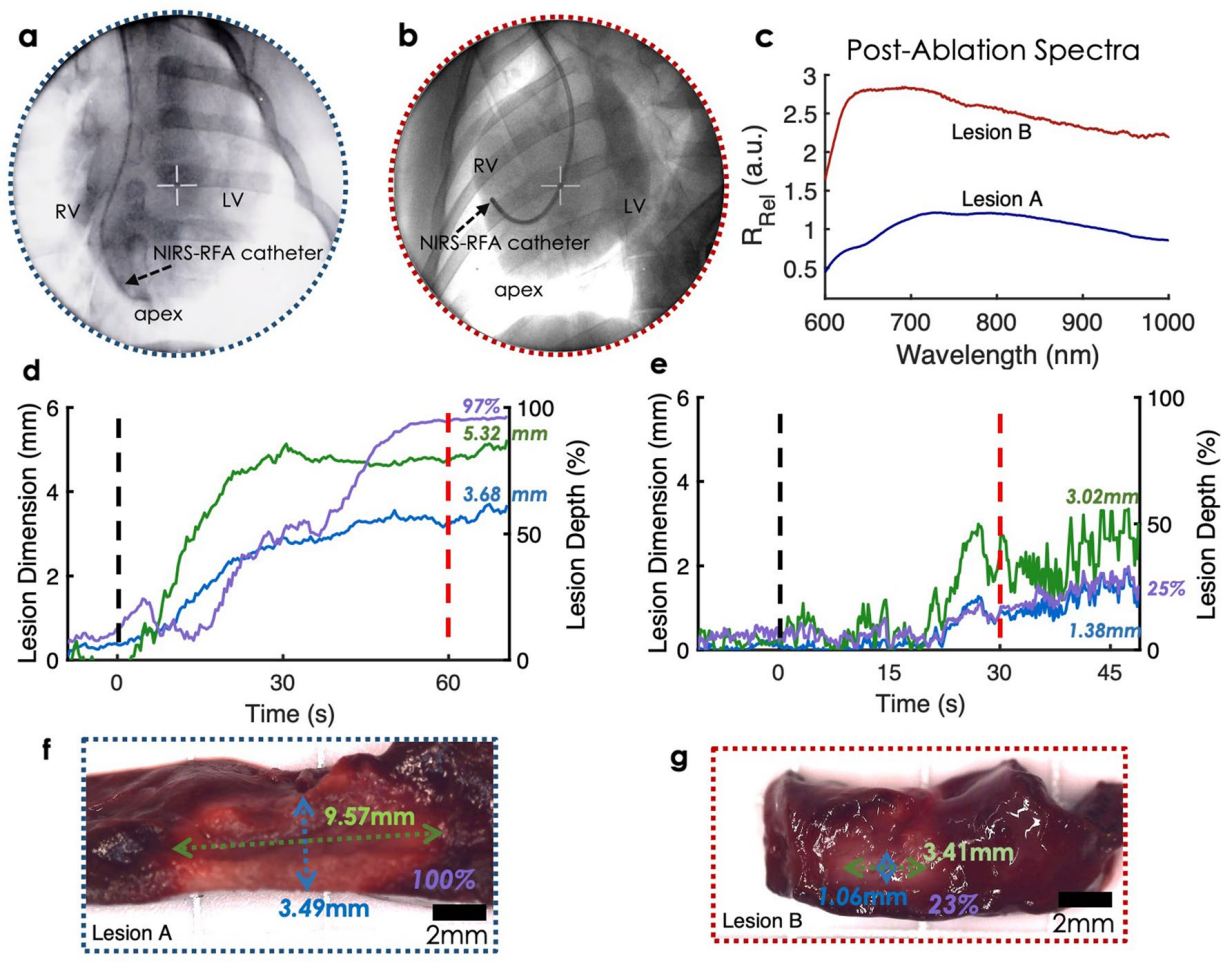
In vivo NIRS assessment of endocardial lesion delivery in pigs. Example measurements are shown for two cases: a transmural (Lesion A) and non-transmural (Lesion B) case. (**a**,**b**) Shows percutaneous navigation of the NIRS-RFA catheter under fluoroscopic guidance for lesion delivery near the apex (Lesion A) and at the RV free wall (Lesion B), respectively. (**c**) Shows final *R*_*Rel*_ spectra measured following the NIRS acquisition of lesion delivery. (**d**,**e**) Depicts time courses of gaussian process regression model predictions derived from final *R*_*Rel*_ spectra for both lesions. Black and red dashes denote the onset and completion. (**f**,**g**) Show corresponding TTC stained cross-sections annotated with actual lesion dimensions.

Healthy pigs underwent endocardial ablation using an open-irrigated NIRS-RFA catheter. Irrigation was fixed at 10 ml/min for all lesions. Lesion A was created near the RV apex shown in Fig. 7a. RF energy was delivered for 60 s with a target power fixed at 25 W. Lesion B was placed at the RV free wall. The ablation target power was set at 20 W for 30 s. Compared to Lesion A (Fig. 7f), size of Lesion B was much smaller (Fig. 7g). In Fig. 7c, post-ablation *R*_*Rel*_ spectra at end of NIRS acquisition highlights spectral morphological differences between transmural lesion and incomplete lesion. Incomplete lesion *R*_*Rel*_ spectra displayed stronger reflectance near 600–750 nm, whereas transmural lesion *R*_*Rel*_ exhibited a right-shifted center of mass; a finding consistent with prior bench-top studies^12^. Local minima observed in ex vivo lesions were not found in either lesion.

In Lesion A, the model showed strong prediction agreement with depth and depth percentage with an error of ~ 0.2 mm and 3%, however, the lesion width was underestimated by 4 mm (~ 44% error). This can be explained by limitations in sampling volume which is a function of the optical fiber configuration and fiber numerical aperture. Another contributing factor may be potential nonlinearities associated with heavier treatment. In Lesion B, our model predicted lesion size with an error of ~ 0.3 mm, ~ 0.4 mm, and 2% for lesion depth, width, and depth percentage, respectively.

## Discussion

In this work, we developed an open-irrigated NIRS-integrated RFA catheter and presented real-time spectroscopic measurements during RF treatment. An optically derived parameter for assessing catheter probe-tissue contact was presented based on endogenous tissue spectral signatures. Additionally, we proposed models for lesion dimension (LD, LW, LD%) estimation in ex vivo swine RV, and real-time monitoring of lesion progression with NIRS on ex vivo and preliminary in vivo healthy pigs. Models based on optically derived parameters offer direct tissue analysis based on the underlying biomolecular composition and ultrastructural makeup. Assessment of lesion formation is a critical factor in determining treatment efficacy but is an unmet clinical need in cardiac electrophysiology. The efficacy of conventional techniques for lesion set validation, such as electrical mapping, can be limited by tissue edema and depolarization, which mimic permanent tissue damage. Auxiliary strategies for determining lesion sufficiency rely on proxies, such as power, contact force, temperature, voltage, time and impedance, to predict lesion progression^2,4,8,45–49^. However, these methods are indirect monitoring and thus may not always capture the direct state of the ablated site. Alternately, magnetic resonance imaging (MRI) has been used to directly evaluate ablation lesions^16,50^. Several groups have demonstrated real-time MRI guidance^51,52^ in cardiac electrophysiology and monitoring of lesion formation during catheter ablation^53,54^. However, MRI imposes additional cost for compatible equipment. Other groups have demonstrated intraoperative tracking of non-irrigated lesion formation using hyperspectral autofluorescence imaging^11,26^, photoacoustic^18,55–57^, and optical coherence tomography (OCT)^15,19,22–25^. Previously, our group demonstrated lesion depth estimation up to 4 mm using NIRS^12^. However, the reported methods above have addressed progression of non-irrigated lesions with a catheter diameter greater than 8-F. Most clinical procedures today use irrigated catheters to generate lesions. Recently, irrigated lesion formation was monitored using fiber-optics based OCT^58^, in vitro, but model for lesion tracking has not been proposed yet. In this study, we developed a novel open-irrigated NIRS-RFA optical catheter and implemented a mathematical model using optically derived features to assess RF treatment quality both ex vivo and in vivo. Such analysis could potentially provide additional wealth of information that can be clinically relevant and improve treatment efficacy. Also, models based on optical parameters offer direct tissue analysis and measurement repeatability as opposed to ablation settings-based treatment indices. In the future, lesion optical indices derived from spectral morphology could be used in feedback control methods for titrating RF energy dose to ensure adequate and sufficient ablation treatment to avoid gaps and electrical signal reconnection.

The model predictions for estimating irrigated lesion dimensions were highly correlated with ex vivo experimental measurements (Fig. 4) up to 6 mm with LOIs. One of the limitations with estimating irrigated lesion progression using conventional methods, such as correlating with tissue temperature, includes cooling of the electrode by blood and irrigation. Often, there exists large temperature variation between electrode and tissue surface^47^. It is doubtful that NIRS optical geometry with a source-detector separation of 0.9 mm samples as deep as 6 mm. Yet, we believe that there exists an analogous relationship between superficial contrast signatures within the first 4 mm, corresponding to the sampling volume^12^, and extent of tissue damage. Thus, tissue surrogate markers help estimate beyond sampling depth. In some cases, the model overestimated lesion dimensions during ablation. An example is shown in Fig. 5b, LW tracking regression model overestimated the width by 1 mm during ablation, but the prediction width dropped to within 0.18 mm after RF offset. The models were trained on *R*_*Rel*_ values 20 s post RF ablation to account for myocardial surface cooling. Lastly, each time course predictions were computed in < 0.1 ms, allowing a real-time, ad-hoc assessment of lesion formation based on optical parameters.

We confirmed that the spectral features of the relative reflectance for a catheter probe in contact with tissue contact in vivo had similar spectral features as it did ex vivo. We demonstrated high statistical significance between that a COI can differentiate catheter-tissue contact and a catheter floating in blood. This parameter distinguished contact within blood-perfused tissue and under the influence of tissue oxygenation and hemodynamics, in vivo.

In addition, our models allow accurate predictions of lesion dimensions in vivo. Lesion depth percentage was assessed to indicate lesion transmurality and estimate lesion size relative to the local wall thickness (< 3% error). In Fig. 7d, a transmural lesion was predicted at 97%. Our training set included only one transmural lesion. Therefore, LD% model could be further improved with the inclusion of additional transmural lesions in the training set. Lesion B LD% prediction was accurate (< 2% error), but a higher variance was observed in Lesion B tracking (Fig. 7e). This can be explained due to the position and orientation of our NIRS catheter. In Fig. 7b, NIRS catheter is curved and placed at the RV free wall. Thus, the quality and angle-orientation of catheter contact may not be as stable as the catheter position for Lesion A, which was placed in the RV apex. Future designs will incorporate a more omnidirectional measurement scheme.

Our work demonstrates the promise of NIRS for assessing dimensions of irrigated RFA lesions. Future studies will be carried out to address current limitations. Firstly, the optical assessment is restricted to acute lesions; scar development from remodeled lesions have yet to be measured. While it is expected that some features will remain predictive, compositional changes may reveal an altered optical-biochemical profile. Chronic, longitudinal studies could give insight into the relationship between acutely measured parameters and durable lesions. Another limitation exists in monitoring catheter maneuverability in vivo. Catheter steerability and orientation tracking could further improve the analysis of the current optical spectral measurement at a wide range of angles in vivo.

## Conclusion

In closing, we developed an integrated NIRS-RFA catheter and demonstrated its utility for estimating the extent of irrigated-lesion formation in real-time within ex vivo and in vivo experiments. Direct optical assessment of the cardiac substrate could aid in supplementing current lesion validation schemes and improving treatment efficacy by offering an independent measurement of lesion sufficiency.

## Supplementary Information

Supplementary Figures.

## Abbreviations

NIRS: Near-infrared spectroscopy
LOI: Lesion optical index
COI: Contact optical index
LD: Lesion depth
LW: Lesion width
LD%: Lesion depth percentage
