# Supplementary material for: Quantification of irrigated lesion morphology using near-infrared spectroscopy
Source: Sci Rep. 2021 Oct 11;11:20160. doi: 10.1038/s41598-021-99725-8 (PMC8505541; doi:10.1038/s41598-021-99725-8)
Supplement: Supplementary file 1 — Supplementary Figures. [file 41598_2021_99725_MOESM1_ESM.docx]

**Supplementary Information: Quantification of irrigated lesion morphology using near-infrared spectroscopy**

Soo Young Park, MSc,^a+^

Rajinder Singh-Moon, Ph.D,^a+^

Haiqiu Yang, MSc,^a^

Deepak Saluja, MD, ^b^

Christine Hendon, Ph.D,^a*^

**Example of recorded lesion optical indices during RF treatment**


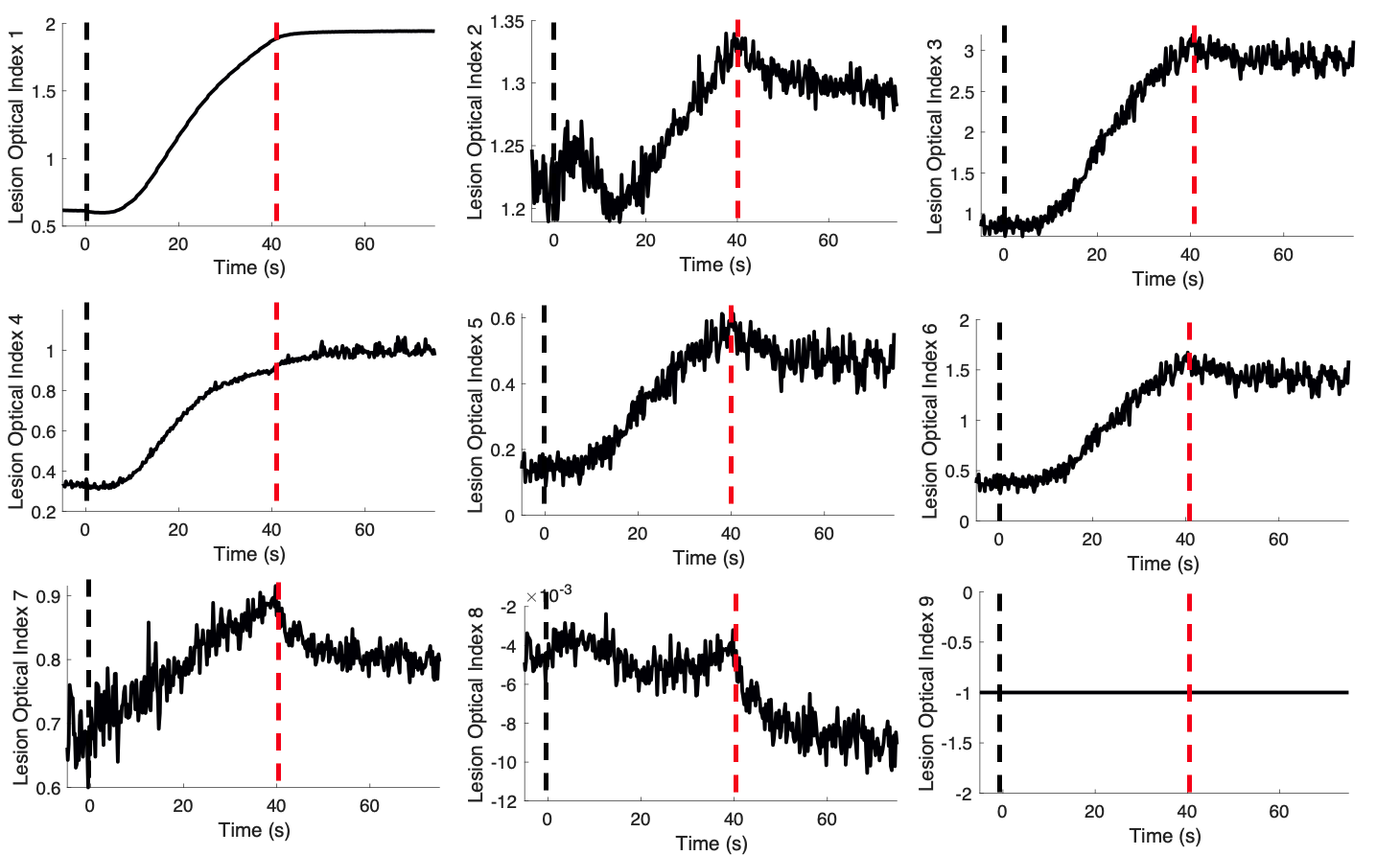


**Supplementary Figure 1.** All 9 lesion optical indices of *Ex vivo* Lesion A in Figure 5.

**Real-time Contact to Non-contact Transition using COI**


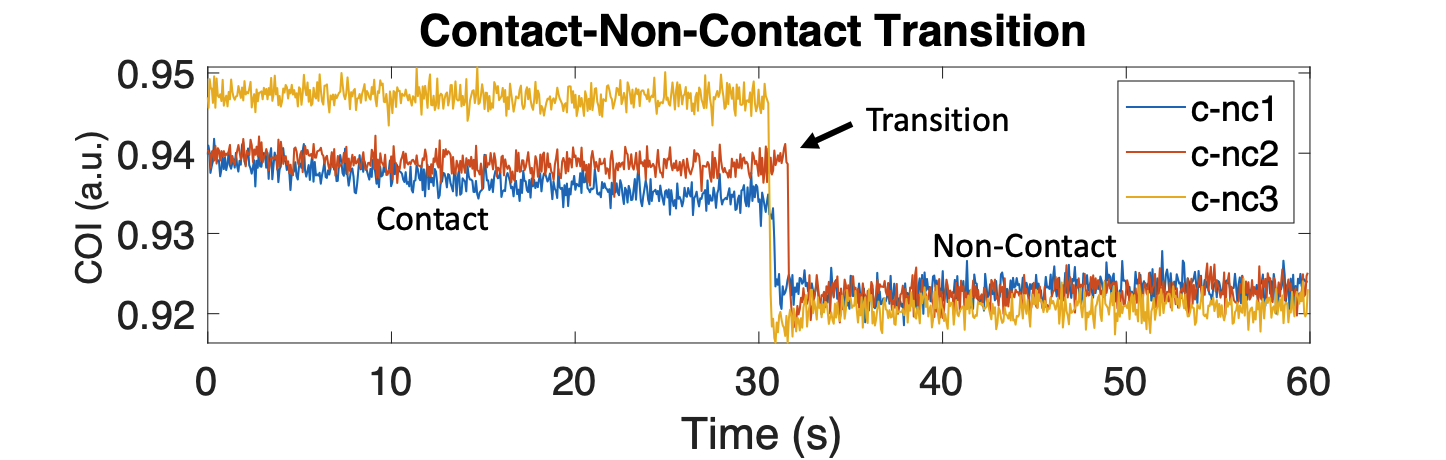


**Supplementary Figure 2.** Real-time contact optical index measurements of contact-Non-contact Transition. Three different cases of real-time monitoring of catheter tip transition: 30 seconds of contact – 30 seconds of non-contact.

**Overlay of bipolar electrogram measurements and R-Rel amplitude variations**


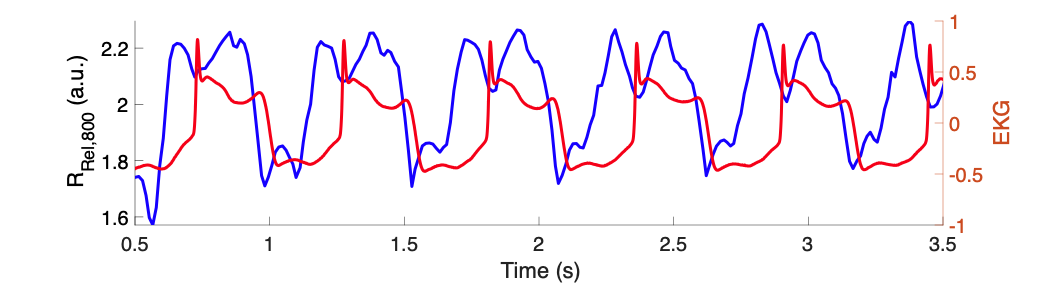


**Supplementary Figure 3.** Comparison of bipolar electrogram measurements and R_Rel_ amplitude variations at 800nm shows corresponding temporal fluctuations over the cardiac cycle and indicates good contact. The recorded bipolar electrograms and R_Rel800nm_ are shown 0.5 seconds after the start of acquisition to show the start of a full cardiac cycle.
